# Explainable phishing website detection for secure and sustainable cyber infrastructure

**DOI:** 10.1038/s41598-025-27984-w

**Published:** 2025-11-25

**Authors:** Tanzila Kehkashan, Maha Abdelhaq, Ahmad Sami Al-Shamayleh, Nazish Huda, Imran Ashraf Yaseen, Abdelmuttlib Ibrahim Abdalla Ahmed, Adnan Akhunzada

**Affiliations:** 1 Faculty of Computing, Universiti Teknologi Malaysia, 81310 Johor Bahru, Malaysia; 2 Department of Information Technology, College of Computer and Information Sciences, Princess Nourah Bint Abdulrahman University, P.O. Box 84428, Riyadh, 11671 Saudi Arabia; 3 Department of Data Science and Artificial Intelligence, Faculty of Information Technology, Al-Ahliyya Amman University, Amman, 19328 Jordan; 4 Faculty of Information Technology, University of Lahore, Sargodha, 40100 Pakistan; 5 Faculty of Information Technology, University of Lahore, Sargodha, 40100 Pakistan; 6 Computer Science Department, Faculty of Computer Science and Information Technology, Omdurman Islamic University, Omdurman, Sudan; 7 College of Computing and Information Technology, Department of Data and Cybersecurity, University of Doha for Science and Technology, Doha, 2444 Qatar

**Keywords:** Machine learning, Phishing website detection, RF, SHAP, URL, Engineering, Mathematics and computing

## Abstract

Phishing is a social engineering attack and a type of cybercrime that is dangerously and constantly on the rise. Phishing attacks can impact various sectors, including governmental, social, financial, and individual businesses. Traditional methods of identifying phishing websites, such as blacklist and heuristic approaches, often fail to provide sufficient protection. Moreover, traditional techniques that combine URLs, webpage content, and external features are time-consuming, require substantial computing power, and are unsuitable for devices with limited resources. Moreover, previous research has often overlooked the critical role of identifying which features are important for detection and their impact on outcomes. Traditional methods might not fully capture the significance of individual features. To overcome this issue, this research applies feature selection techniques, specifically shapley additive explanations, with each model based primarily on the URL to improve the detection process. A dataset with over 11000+ URLs and 30 varied features of the ”Phishing Website Detection” was applied from the Kaggle repository. Then, the models, namely support vector machine (SVM), random forest (RF), decision tree (DT), logistic regression(LR), and K-nearest neighbor, were trained and tested. Each model used shapely additive explanations (SHAP) to improve precision and interpretability by highlighting the most important features. It was tested using some key performance metrics such as accuracy, precision, recall, and F1 score. Compared to all the models that were tested, this random forest model indicates 97% accuracy. The proposed system offers an overall and interpretable solution for phishing detection that contributes to a safer digital environment.

## Introduction

Phishing is a widespread cyber attack based on social engineering where attackers trick individuals into disclosing sensitive data like usernames, passwords, and financial information. Phishing is a widespread threat across various sectors like government departments, banks, social media websites, and personal users^1^. Due to the fast expansion of internet services like mobile apps and cloud computing, the rate of online transactions and electronic communications has grown significantly^2^. As a result, phishing has become a significant cybersecurity issue, with the attackers constantly evolving their methods in order to bypass current security measures^3^. Phishing sites, which typically depend on misleading URLs to entice victims, are one of the most popular attack vectors for online fraud^4^. Malicious links are typically disseminated through email, SMS, and social media sites, hence turning phishing into an ongoing and ubiquitous threat^5^. It has been reported that over 80% of firms experience a phishing attack every year, which leads to significant financial and operational consequences^6^. The increasing sophistication and magnitude of such attacks emphasize the need for detection methods to be precise, explainable, and cost-effective.

Traditional detection methods like blacklists and heuristic-based have been widely used^7^. Nevertheless, these approaches are narrow in scope and ineffective for extremely dynamic and new phishing attacks^8^. More sophisticated approaches based on URL examination, web page content, and extrinsic features have been brought up, yet they are computationally costly and unsuitable for low-capacity devices^9^. Feature selection methods, such as Particle Swarm Optimization and Information Gain, have been used to enhance efficiency, but they are still limited by long computation times and partial feature identification^10^.

Even with these improvements, phishing detection remains challenging. Attackers use more evasion techniques, making static detection systems useless^11^. Present methods cannot also handle zero-day attacks through dependence on stale or static features^12–14^. In addition, traditional feature selection techniques often do not account for the heterogeneity of phishing URLs, thus compromising model robustness and generalizability^15^. These shortcomings highlight the importance of phishing detection systems that not only work well but also are explainable and sustainable.

To tackle these issues, this research proposes an explainable phishing detection framework that combines Shapley Additive Explanations (SHAP) with supervised machine learning (ML) models. By exploiting URL-based inspection and SHAP-guided feature selection, the suggested solution improves interpretability while being computationally efficient. The novelty of this work is in integrating explainable feature selection with state-of-the-art ML classifiers to achieve both excellent predictive performance and human-interpretable insights, thus making progress in the design of useful and sustainable phishing detection systems.The goals of this work are as follows:

i. To establish a strong method for phishing website detection using URL-based features with enhanced accuracy and efficiency.
ii. To integrate SHAP with ML models like SVM, KNN, RF, DT, and LR for feature interpretation and better model explainability.
iii. To examine the effect of SHAP-based feature selection on the performance of several supervised ML models.

### Contributions of this study

This study provides a number of contributions to phishing detection. First, it proposes an innovative explainable detection approach that combines SHAP and supervised machine learning algorithms for phishing website detection. Second, it improves feature interpretation through the identification of the most impactful URL-based features, thus yielding actionable insights for cybersecurity professionals. Third, it presents a benchmark testing by means of systematic experimentation on the Kaggle Phishing Website Detection dataset, with over 11,000 URLs and 30 different features. Fourth, the research shows notable performance improvement, with the Random Forest model augmented with SHAP exhibiting the best accuracy of 97%, surpassing other classifiers when it comes to precision, recall, and F1-score. Lastly, the suggested solution is focused on practical usability in that it is interpretable, cost-effective, and deployable in real-world resource-limited environments.

The rest of the paper is structured as follows. Section 2 discusses a review of related work in phishing detection, including recent developments and lacunae. Section 3 describes the research methodology, including data sources, feature extraction, and ML classifiers. Section 4 discloses the experimental results, comparing the proposed method with current ones and exploring the findings. Section 5 presents the implications of the findings, and Sect. 6 concludes the research by highlighting the greatest contributions and areas for future study.

## Literature review

To conduct a literature review, this study follows the framework proposed by Saied et al.^16^, which provides guidelines for transparent and reproducible research analysis. Relevant studies on ML, deep learning (DL), and hybrid approaches for phishing detection were selected and evaluated based on dataset characteristics, feature extraction methods, model architecture, and reported performance metrics. This methodology ensures consistent comparisons, highlights gaps in prior work such as limited interpretability, and establishes a solid foundation for proposing the RF model enhanced with SHAP.

### Blacklist and whitelist techniques

Traditional phishing detection initially relied on blacklists and whitelists to identify malicious websites^17–19^. Blacklists maintain a repository of known phishing URLs and flag websites accordingly. While straightforward, blacklists are inherently reactive, unable to detect new or zero-day phishing sites. Whitelists, which permit access only to verified legitimate websites, face similar limitations due to attacker evasion strategies. Both methods are further constrained by the dynamic nature of phishing campaigns, as they require continuous updates that are often slow, manual, and resource-intensive^20,21^. These approaches provided a foundation for early phishing detection but proved insufficient against adaptive adversaries. Their reactive design means they fail to anticipate evolving threats, and their reliance on human updating undermines scalability. As phishing campaigns became more automated and sophisticated, the shortcomings of these methods motivated the transition toward data-driven, automated, and more adaptive solutions.

### Traditional techniques for phishing detection

Early heuristic-based systems attempted to go beyond blacklists by relying on manually defined rules. Such methods assessed suspicious URL structures, domain names, and the presence of unusual characters or tokens^19–21^. Heuristic rules had the advantage of being lightweight and independent of large datasets, but they frequently produced high false positive rates and lacked the robustness needed for evolving phishing campaigns. For instance, attackers could easily bypass heuristics by slightly altering domain names or obfuscating malicious indicators. Critically, these approaches offered little to no interpretability. Security analysts could see that a website was flagged as malicious but lacked insight into why. This “black-box flagging” not only reduced trust in automated systems but also limited their adoption in organizational security infrastructures^22,23^. As phishing attacks began to imitate legitimate websites with near-perfect fidelity, heuristic approaches became increasingly inadequate. These limitations directly motivated the shift toward ML and, more recently, explainable AI (XAI), where interpretability is not optional but essential for adoption in high-stakes cybersecurity contexts.

### ML techniques for phishing detection

Machine learning rapidly emerged as a dominant solution to phishing detection, offering adaptability, pattern recognition, and the ability to learn from large datasets. ML methods can analyze URL structures, webpage content, and external metadata to distinguish legitimate sites from phishing attempts^24^. Among these, URL-based approaches remain particularly attractive due to their computational efficiency and reduced dependence on resource-heavy web content parsing^25,26^. Hybrid methods that combine URL features with content and external metadata have achieved higher accuracy, as they integrate diverse sources of information^27,28^. Ensemble learning further strengthens these methods. For example, RF, KNN, and ANN combinations improve resilience and adaptability^29–31^. Similarly, paired classifiers such as SVM-KNN and LR-DT with AdaBoost have demonstrated better generalization to unseen attacks^32,33^. Ahmad et al.^34^ reviewed AI-driven phishing detection systems and emphasized that ensembles integrated with real-time threat intelligence significantly improve adaptability against fast-evolving phishing campaigns. However, most ML-based works continue to rely on extensive manual feature engineering, which is resource-intensive and prone to overlooking subtle but critical signals. Moreover, while accuracy rates are frequently reported, interpretability remains underexplored. In practice, decision-makers must understand why a model classifies a site as phishing before acting on it. This gap underlines the importance of XAI-based feature selection methods such as SHAP, which can provide interpretable explanations while maintaining predictive strength.

### DL techniques for phishing detection

Deep learning has brought major advances by enabling automatic feature extraction from raw data. CNNs capture spatial and sequential structures in URLs, reducing reliance on manual engineering^35–37^. LSTMs and other RNNs excel at modeling sequential dependencies, particularly valuable in identifying obfuscated URLs that mimic legitimate naming patterns. Incorporating external signals such as SSL certificates, registrar details, and domain age further strengthens detection^38^. Hybrid DL architectures, combining CNN and LSTM layers, have produced state-of-the-art results in detecting increasingly sophisticated phishing techniques^39^. Despite these strengths, DL approaches present significant challenges. They require large labeled datasets and substantial computational resources, which may not be feasible for small organizations or low-resource environments. Moreover, interpretability remains a major barrier: deep models often function as “black boxes,” making their decisions difficult to justify in operational or legal contexts^40,41^. Insights from other domains illustrate possible directions. For instance, transformer-based DL architectures have advanced fields such as plant disease detection^42^ and micro-expression recognition^43^, demonstrating how self-attention mechanisms capture complex dependencies efficiently. In fraud detection, transformer-enabled recruitment fraud detection^44^ and AI-driven IoT security frameworks^45^ show the utility of advanced DL in high-risk contexts. Similarly, CoAtNet-based medical imaging^46^, deep CNN gesture recognition^47^, and hybrid ML–DL video captioning studies^48^ further highlight the scalability and robustness of attention-driven models. Nevertheless, the trade-off between performance and explainability remains unresolved, reinforcing the need for interpretable and lightweight alternatives.

### Quantitative and comparative techniques

Comparative studies benchmark different phishing detection models using quantitative metrics such as accuracy, precision, recall, and F1-score^28,29^. RF frequently outperforms other classifiers due to its ensemble structure and robustness to noisy features, while hybrid models combining classifiers and feature sources achieve stronger generalization^31,32,49^. Yet, a critical observation emerges: performance metrics alone are insufficient. High accuracy may obscure issues such as poor interpretability, long training times, or poor adaptability to unseen zero-day attacks. Several comparative analyses now advocate for a balance of accuracy, efficiency, and explainability. Without this, high-performing models risk remaining academic exercises, rarely adopted in operational cybersecurity infrastructures. This aligns with calls in other domains for explainable solutions, as seen in finance, where XAI frameworks such as SFIX (Scalable Financial-oriented Interpretable eXplanation)^50^ and systematic reviews of explainable AI in financial applications^51^ highlight the centrality of interpretability for adoption. Such parallels emphasize that phishing detection research must equally embrace XAI if it is to transition successfully into practice.

###  Feature engineering for phishing detection

**Fig. 1:**
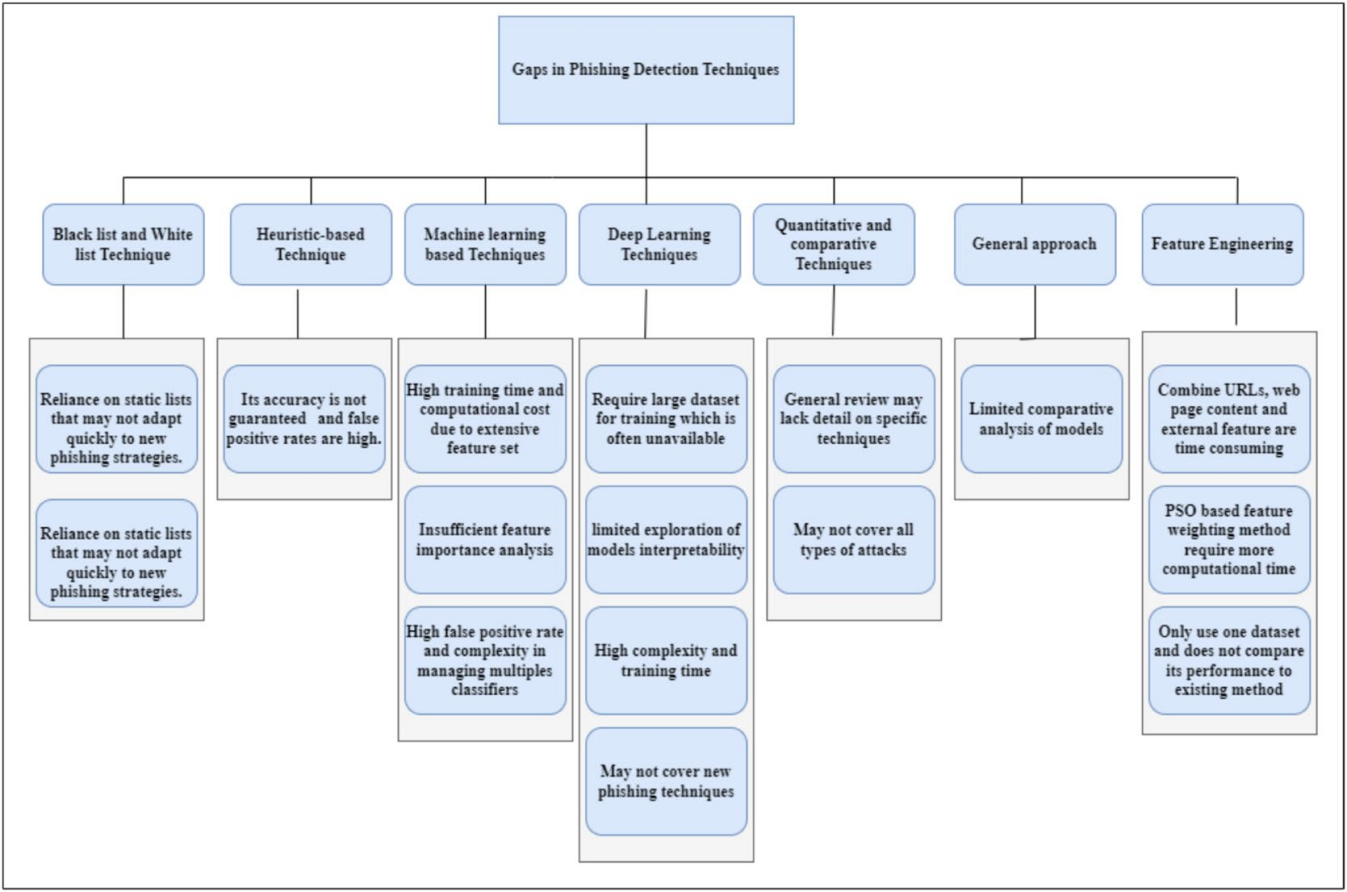
Gaps in Phishing Detection Techniques: Challenges across blacklists, heuristics, feature engineering, interpretability, and computational constraints.This diagram summarizes the current limitations and unexplored areas in phishing detection methodologies. It highlights key gaps in existing models, such as lack of feature generalization, absence of explainability, and limitations in adapting to real-time phishing campaigns.

Feature engineering remains central to phishing detection, influencing both performance and interpretability. Approaches such as genetic algorithms, permutation importance, and metaheuristics have been applied to optimize features and reduce computational costs^52–56^. More recent work emphasizes interpretable methods like SHAP, which explain predictions by quantifying each feature’s contribution^57–60^. Several innovative strategies combine advanced feature selection with resampling and balancing methods. Examples include CatBoost with SMOTE-Tomek balancing, BERT-based embeddings, BMEO-KNN, and document-term matrix feature extraction^61–65^. These approaches show that model transparency and stability improve when robust feature engineering is combined with interpretability tools. In addition, ensemble-based feature engineering methods are emerging as powerful alternatives. For example, ensemble learning for financial data classification has successfully leveraged encrypted datasets while maintaining interpretability citeKucur2025AuditOpinions, providing a methodological parallel for phishing detection research. Despite progress, persistent gaps remain. Many approaches still depend on handcrafted features, which are vulnerable to adversarial manipulation. Computational costs remain high, limiting practical deployment on low-resource devices. Most importantly, interpretability is often an afterthought rather than a central design criterion. Figure 1 summarizes these limitations, highlighting the need for models that combine efficiency, transparency, and strong generalization. Building on this critical evaluation of existing methods, this study proposes a SHAP-based ML framework that efficiently identifies key URL features, ensures interpretability, and achieves high predictive performance while remaining suitable for deployment in resource-constrained cybersecurity infrastructures.

## Methodology

This paper suggests a novel approach to detecting phishing websites using supervised ML models combined with SHAP to incorporate explainability in features and improve detection accuracy. The approach is designed to fill the gap between high-performing detection and feature importance understanding, which is vital in real-world cybersecurity practice. The architecture of the framework consists of five principal components: baseline comparison, model selection, data gathering, preprocessing and model design, and experiment realization.

### Baseline method

**Fig. 2:**
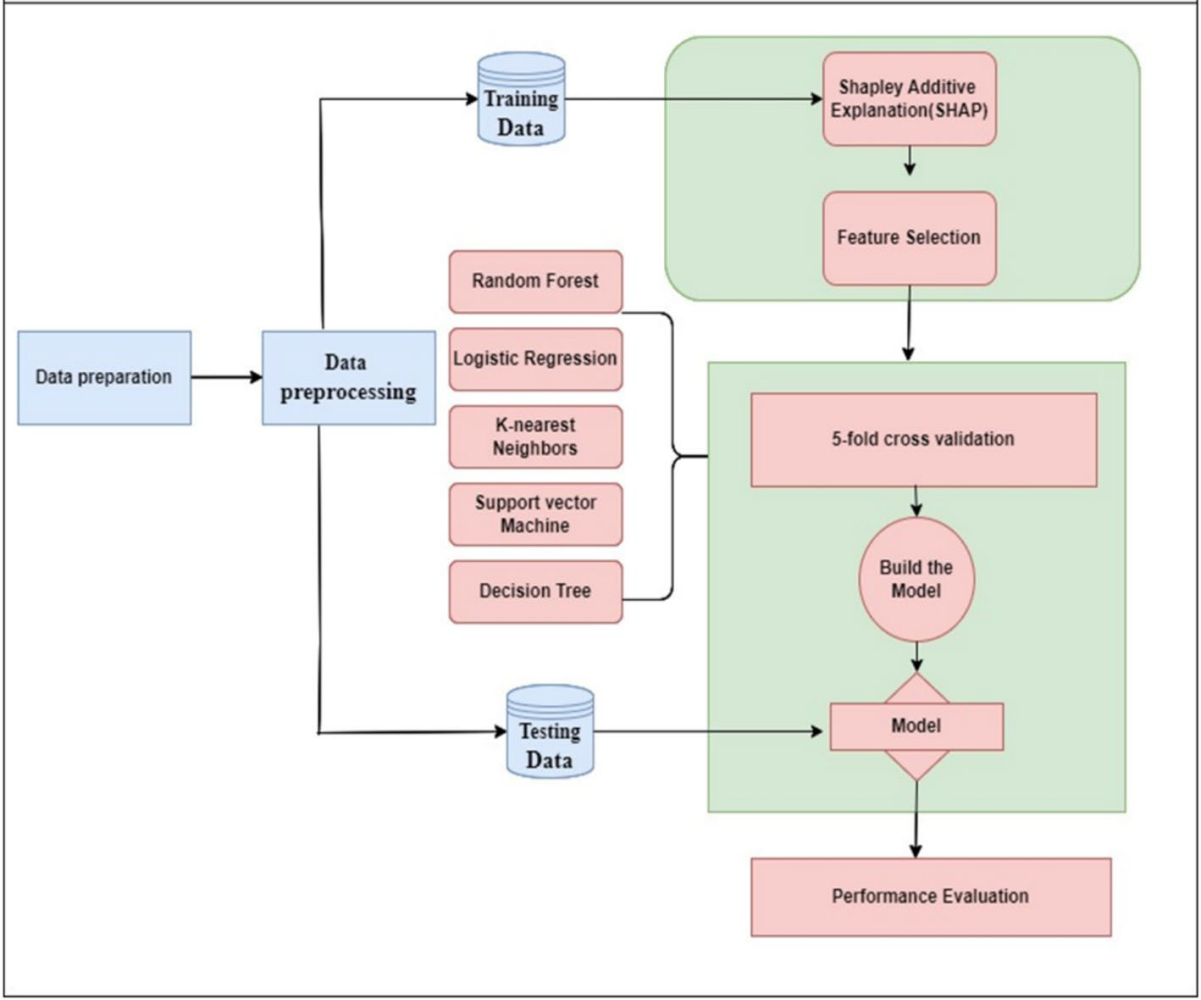
Proposed Methodology Diagram.This figure presents the end-to-end flow of the proposed phishing detection framework. It includes data collection, preprocessing, feature selection, model training, evaluation, and explainability phases, organized in a sequential and modular fashion.through ensemble averaging.

The baseline for this study is the work of^66^, who introduced a ML solution for phishing detection based on URL-based features. The baseline used models like RF and SVM to identify URLs as phishing or not, with the primary aim of detection accuracy. Although the baseline worked well, the baseline was not interpretable and did not offer feature contribution insights. Our suggested methodology builds on this baseline by incorporating SHAP for feature importance analysis while preserving performance in detection without sacrificing interpretability. Figure 2 demonstrates the block diagram of our suggested methodology, indicating the flow of data collection, preprocessing, model learning, SHAP-based feature selection, and performance testing.

### Models selection

Our approach utilizes several supervised learning models for high-quality detection and comparative evaluation. RF, SVM, DT, LR, and KNNs are used as the chosen models. RF combines several DTs by majority voting to increase accuracy and avoid overfitting. SVM tackles high-dimensional data efficiently with the help of hyperplanes to classify classes. KNN predicts a data point according to the majority vote of its nearest neighbors, whereas LR estimates the probability of binary classes with a logistic function. DTs divide datasets by feature values to create a tree-like structure, optimizing homogeneity within segments.

Each model is paired with SHAP, which computes feature contributions to the end predictions, which increases interpretability and sheds light on which features have the greatest impact on classification decisions. This addresses some of the criticisms of past approaches cited by the reviewers, notably the absence of feature-level interpretability and real-world insight into detecting phishing.

### Data collection

The dataset used in this research was obtained from the UCI Machine Learning Repository, originally contributed by Mohammad et al.^63^. It is publicly available at: https://archive.ics.uci.edu/ml/datasets/Phishing+Websites. This benchmark dataset has been widely used in phishing detection research, ensuring reproducibility and comparability of results across studies. The dataset contains 11,055 website records labeled as phishing (1) or legitimate (− 1), with 30 handcrafted features extracted from website URLs and related attributes. Some of the most relevant features include: *UsingIP*, *LongURL*, *ShortURL*, *Symbol@*, *PrefixSuffix-*, *SubDomains*, *HTTPS*, *DomainRegLen*, *Favicon*, *NonStdPort*, *RequestURL*, *AnchorURL*, *IframeRedirection*, *AgeofDomain*, *DNSRecording*, *WebsiteTraffic*, *PageRank*, and *LinksPointingToPage*. The dataset provides a diverse representation of URL patterns and domain-related attributes, allowing models to learn discriminative characteristics of phishing websites compared to legitimate ones. Its capacity is large enough to support vigorous training and validation, and its status as a benchmark dataset increases the certainty of comparative assessment with alternative state-of-the-art approaches.

### Preprocessing

Preprocessing is a crucial process to prepare the dataset for effective training of the model and accurate phishing detection. The initial dataset contains over 11,000 URLs with 30 attributes including varied URL features, web content features, and domain features. Data cleaning is initially performed to remove duplicates and unwanted entries in order to preserve the purity of the dataset. Categorical data are encoded numerically, and feature scaling is employed to normalize the range of values over all features to prevent bias towards ranges with high numbers. Pre-processing the dataset into training (80%) and testing (20%) sets prevents weak evaluation while ensuring that there is a sufficient amount of data for model training. URL-specific pre-processing includes tokenization of the URL strings and encoding them numerically in formats suitable for the ML algorithms. Besides, SHAP has been applied to feature selection in order to identify the most influential attributes and eliminate less informative ones. This not only maximizes model performance but also enhances interpretability as it indicates how features contribute to phishing detection results. We empirically chose the top 15 features according to SHAP ranking, since this achieved the best trade-off between interpretability and model accuracy.

### Model architecture

The suggested methodology utilizes various supervised learning models, i.e., RF, SVM, DT, LR, and KNNs, with all of them combined with SHAP to offer feature-level interpretability. The input features, like HTTPS usage, Domain Registration Length, URL length, and special symbols, are passed through the models. SHAP values are calculated for every feature to measure their contribution to the predictions made by the model, allowing a transparent assessment of importance of features.

The RF classifier combines the predictions of many DTs to improve robustness and avoid overfitting. Each tree is trained on a random subset of data and, at each node, a random subset of features is used for splitting. The overall prediction is the majority vote across all trees, represented mathematically as:

1 $$\begin{aligned} F(z) = \frac{1}{M} \sum _{j=1}^{M} g_j(z) \end{aligned}$$

where *M* is the total number of trees, and $g_j(z)$ denotes the prediction of the *j*-th tree.

SVMs are applied for classification in high-dimensional feature spaces, separating categories with optimal hyperplanes. The decision function for SVM is given as:

2 $$\begin{aligned} D(u) = \alpha \cdot u + \beta \end{aligned}$$

where $\alpha$ represents the weight vector, *u* is the input feature vector, and $\beta$ is the bias term.

The KNN algorithm determines the class of a sample based on the majority label among its closest neighbors, typically measured by the Euclidean distance:

3 $$\begin{aligned} \delta (p, q) = \sqrt{\sum _{k=1}^{d} (p_k - q_k)^2} \end{aligned}$$

where *p* and *q* are feature vectors, and $p_k, q_k$ denote the values of the *k*-th feature. In this study, $K=5$, indicating that the five nearest samples are considered for classification.

DTs partition the dataset recursively by splitting on attribute values, forming a tree where internal nodes correspond to decision criteria, edges represent outcomes, and leaf nodes provide the predicted class. LR estimates the probability of binary outcomes using the logistic function:

4 $$\begin{aligned} \Pr (y=1|u) = \frac{1}{1 + e^{-(\alpha \cdot u + \beta )}} \end{aligned}$$

where $\alpha$ are the feature coefficients and $\beta$ the intercept term.

SHAP values are calculated for all models, enabling interpretation by quantifying the contribution of each feature to the final prediction.

### Implementation details

The database has more than 11,000 URLs with binary labels for phishing (1) or legitimate (− 1) web pages. Post-preprocessing, features are domain-specific parameters like URL length, special characters, use of HTTPS, and redirection flags. Categorical features are converted into numerical format by encoding and normalization to gain equal scaling for models. The database is split between training (80%) and testing (20%) sets, and five-fold cross-validation is used to gain reliable evaluation and reduce overfitting. The computation environment consists of Windows 10, 2.4 GHz processor, and Google Colab GPU, which offer adequate resources to train the model. Table 1 gives an overview of components and experimental setup.

**Table 1:** Components Description.This table outlines the dataset specifications and experimental environment used in the study. It details the source of data, dataset size, number of features, classification type, and system configurations such as processor, OS, and computational resources.

Components	Description
Dataset used	Kaggle / UCI repository
Training and testing split	80/20
Classes	Binary (legitimate/phishing)
No. of samples	11,000 URLs
No. of features	30
System used	Windows 10
GPU	Google colab GPU
Processor	2.4 GHz processor

SHAP-based feature ranking is performed across all models to identify the most influential features, improving model transparency and enabling interpretable predictions. The methodology ensures that the final prediction is a binary classification of phishing or legitimate websites, combining robust ML performance with feature-level interpretability. The integration of SHAP, careful preprocessing, and ensemble techniques results in a methodology that is accurate, efficient, and applicable in real-world phishing detection scenarios. Algorithm 1 formally summarizes the SHAP-enhanced RF training and evaluation process.

**Algorithm 1:**
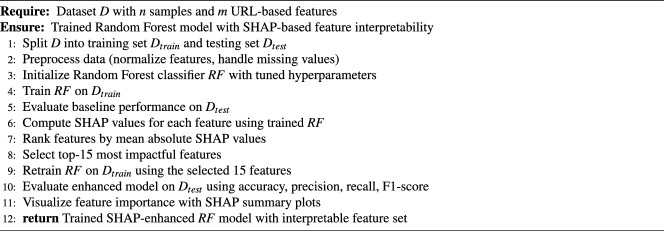
SHAP-enhanced random forest for phishing detection

## Experiments and results

In this work, we present a systematic evaluation of the new ML–based approach for phishing website detection. Experimental results demonstrate that our approach consistently obtains outstanding classification performance on different evaluation metrics and clearly surpasses some prominent methods reported in recent literature. This section consists of five primary components: model performance, ablation studies, comparison with competing detection models, qualitative explanation, and overall discussion. Each of them has critical observations on the strengths and weaknesses of the system, along with effectiveness and areas of improvement. The results exhibit the performance and reliability of the sophisticated framework to identify genuine and phishing web pages with accuracy. Through integrating high performance with explainability, the study makes a useful contribution to the advancement of realistic and sustainable phishing detection systems.

### Performance analysis of proposed method

We first compare the performance of five popular supervised learning classifiers to determine the best phishing website detection classifier. Table 2 reports the comparative analysis in terms of F1-score, precision, recall, and accuracy.

**Table 2:** Performance Analysis of Proposed Methodology with SHAP (in %)This table presents the performance metrics of various ML algorithms when enhanced with SHAP. It demonstrates the improvements in interpretability and performance across precision, recall, F1-score, and accuracy.

Algorithms	F1-score	Precision	Recall	Accuracy
KNN	94.9	94.6	95.1	94.3
SVM	94.2	92.9	95.5	93.5
LR	94.1	92.9	95.3	93.3
DT	96.4	96.7	96.1	96.0
RF	97.3	97.0	97.6	97.0

The KNN model achieved an F1-score of 94.9, precision of 94.6, recall of 95.1, and accuracy of 94.3. The SVM achieved an F1-score of 94.2, precision of 92.9, recall of 95.5, and accuracy of 93.5. LR reached an F1-score of 94.1, precision of 92.9, recall of 95.3, and accuracy of 93.3. The DT model obtained an F1-score of 96.4, precision of 96.7, recall of 96.1, and accuracy of 96.0. Finally, the RF model outperformed all others, achieving an F1-score of 97.3, precision of 97.0, recall of 97.6, and accuracy of 97.0. These results provide strong evidence that RF is the most suitable supervised learning algorithm for phishing website detection in our setting.

**Fig. 3:**
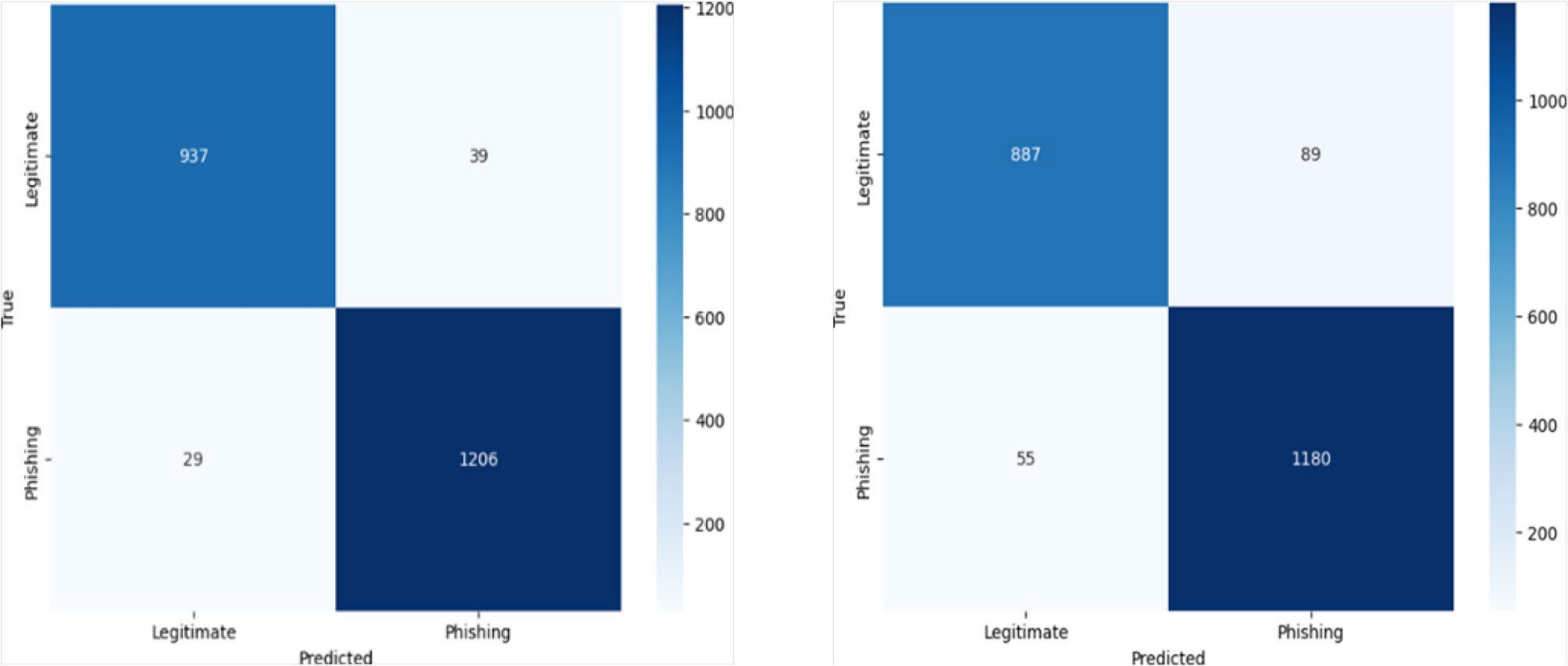
RF model Confusion Matrix (Right) and SVM model Confusion Matrix (Left).A side-by-side comparison of the confusion matrices for the SVM and RF classifiers. The matrices illustrate the models’ classification performance, showing true positives, false positives, true negatives, and false negatives.

**Fig. 4:**
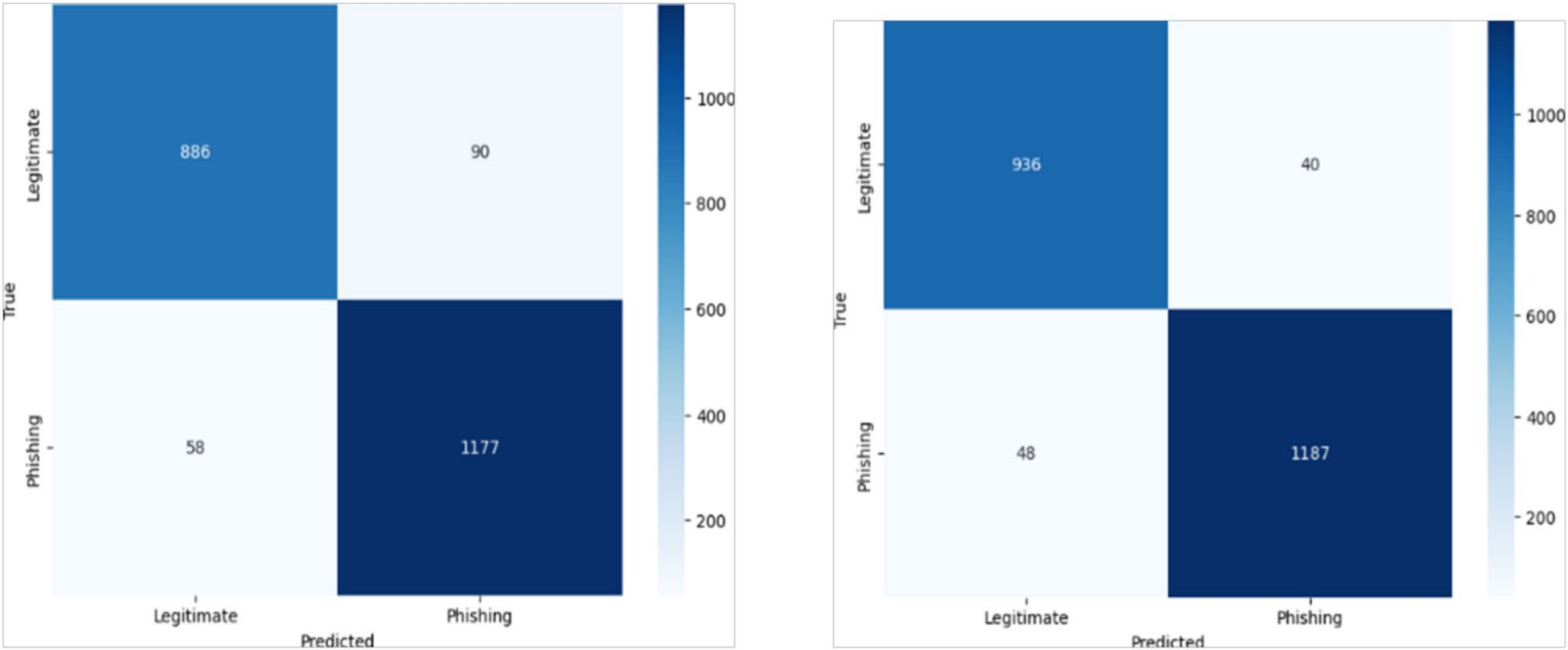
LR model Confusion Matrix (Right) and DT model Confusion Matrix (Left).This figure presents the confusion matrices for the DT and LR models. It visually compares the predictive accuracy and misclassification rates for both classifiers.

**Fig. 5:**
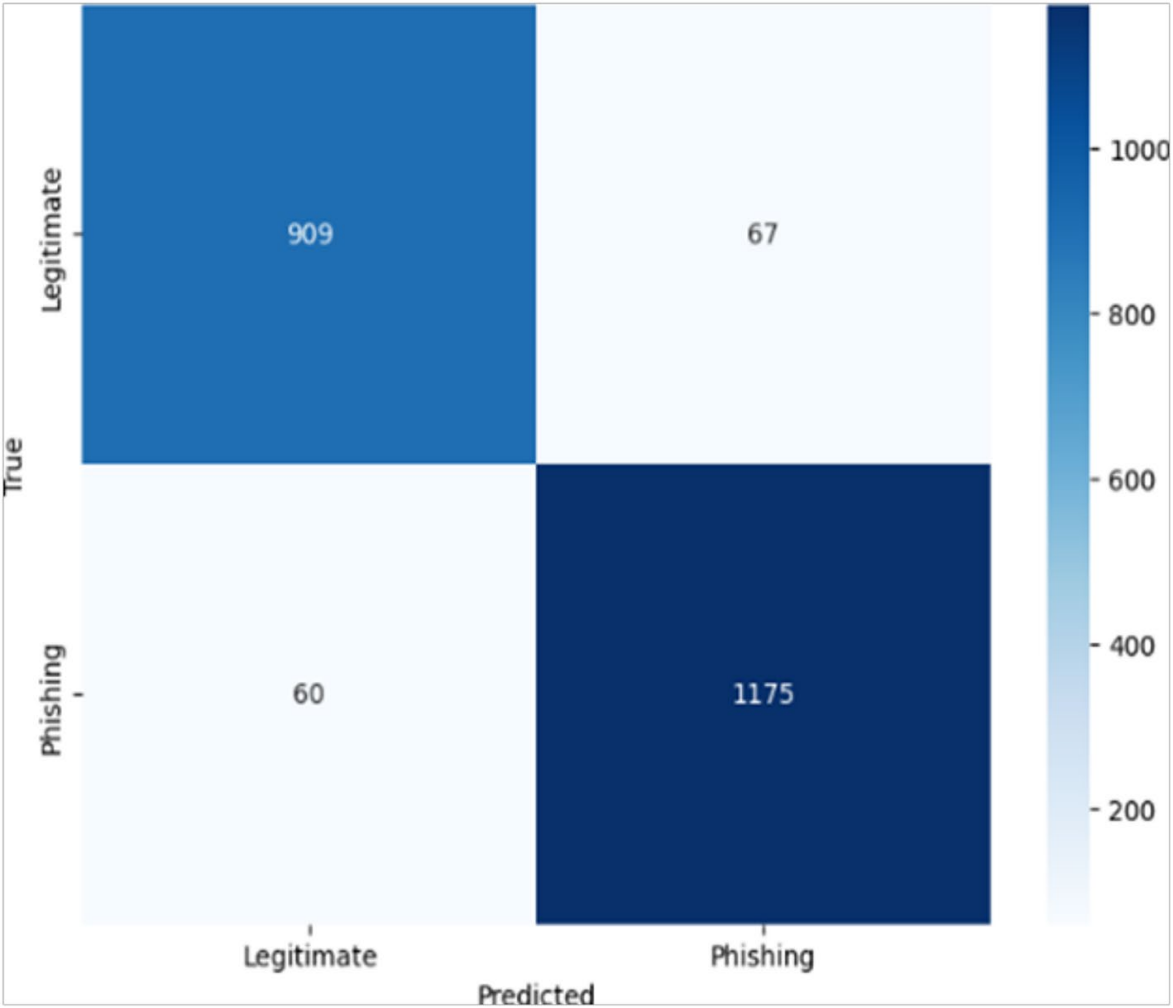
KNN model Confusion Matrix.The confusion matrix of the KNNs model is shown, highlighting its classification performance in detecting phishing instances based on proximity-based feature matching.

**Fig. 6:**
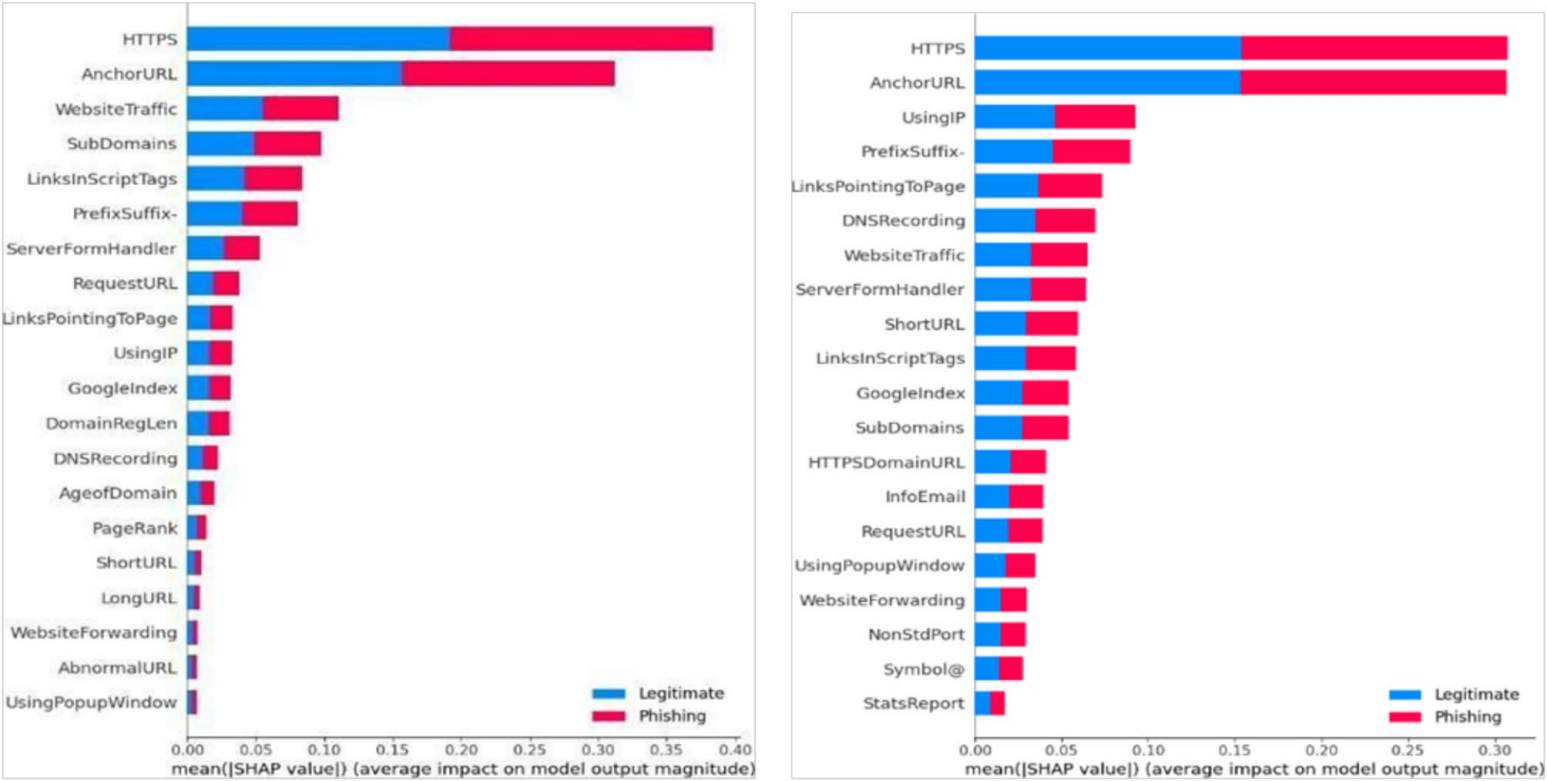
Feature importance ranked by SHAP for RF (Right) and SVM (Left).SHAP value plots for SVM and RF models, ranking the features based on their contribution to the final prediction. These visualizations enhance interpretability by explaining the impact of each feature on the model’s decisions.

**Fig. 7:**
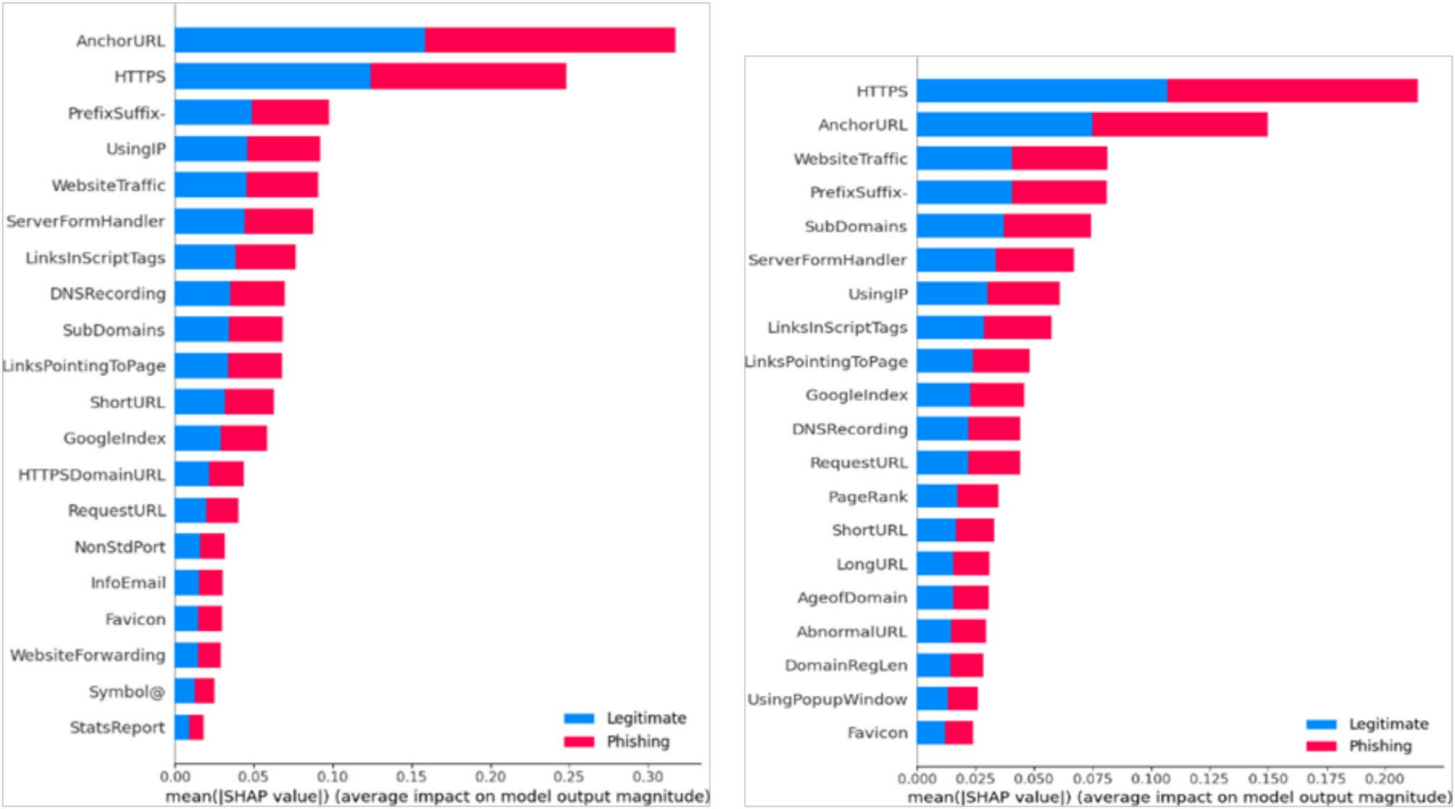
Feature importance ranked by SHAP for LR (Right) and KNN (Left).This figure displays SHAP feature rankings for the KNN and LR models. It helps interpret which input variables most influenced the prediction in both classifiers.

**Fig. 8:**
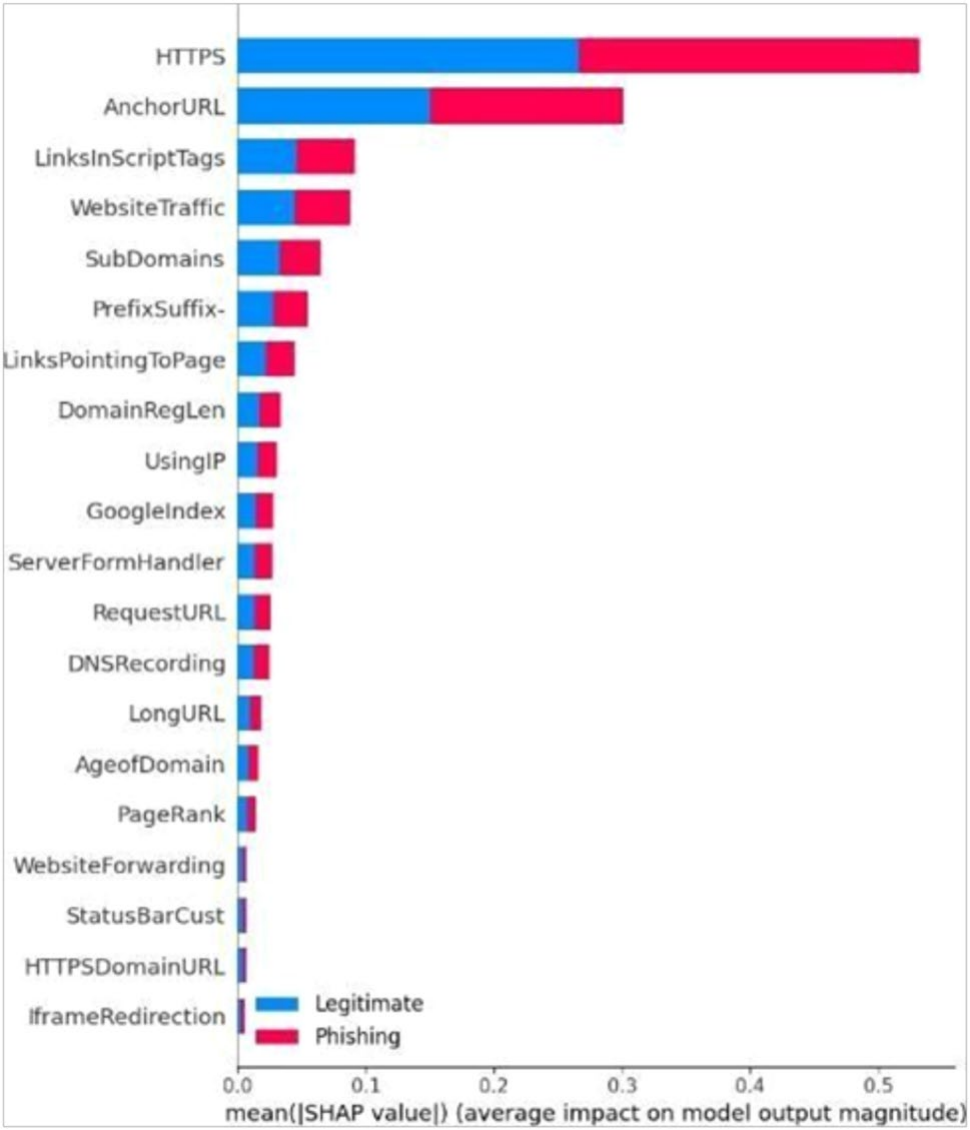
Feature importance ranked by SHAP for DT.The SHAP plot illustrates the top-ranked features influencing the DT model’s predictions. This improves the transparency and trustworthiness of the model by identifying how feature values drive classification.

Analyzing the confusion matrices, in Fig. 3: the RF model correctly identified 937 true negatives and 1206 true positives, with only 39 false positives and 29 false negatives and the SVM achieved 887 true negatives and 1180 true positives, alongside 89 false positives and 55 false negatives. In Fig. 4, LR produced 886 true negatives and 1177 true positives, but slightly higher misclassifications with 90 false positives and 58 false negatives and the DT yielded 936 true negatives and 1187 true positives, with 40 false positives and 48 false negatives. Finally, the KNN (Fig. 5) model achieved 1157 true negatives and 909 true positives, while recording 67 false positives and 60 false negatives. These results confirm that although all models demonstrated competitive performance, the RF classifier consistently achieved the best balance across all metrics (Fig. 6).

To further enhance interpretability, SHAP was applied to evaluate the relative importance of each feature in the classification process. SHAP provides a transparent mechanism to understand feature contributions to model decisions, thereby increasing trust in predictions. Applying SHAP across all models revealed the most influential features that drive accurate phishing detection. The summary plots visualize these contributions by showing the relationship between feature importance (y-axis) and SHAP values (x-axis), clarifying how individual features affect predictions. This interpretability strengthens the transparency and robustness of the detection system.

### Ablation studies

The ablation study evaluates the effect of incorporating SHAP on model performance. Table 3 reports the baseline results without SHAP. Comparing these results with those in Table 2 demonstrates the clear advantage of using SHAP-enhanced feature importance.

**Table 3:** Models Performance Metrics Without SHAP (in %). This table shows the baseline performance of ML models without incorporating SHAP explainability. It serves as a comparison point to highlight the benefits gained through SHAP-based interpretability in the previous table.

Algorithms	F1-score	Precision	Recall	Accuracy
KNN	88.0	87.0	89.0	86.0
SVM	87.0	85.0	88.0	86.0
LR	86.0	84.0	88.0	85.0
DT	91.0	90.0	91.0	90.0
RF	92.0	91.0	93.0	90.0

For example, the RF classifier achieved 90.0% accuracy without SHAP, which increased to 97.0% after SHAP-based feature refinement. Similar improvements were observed across other models: KNN (Fig. 7) improved from 86.0% to 94.3%, SVM from 86.0% to 93.5%, LR (Fig. 7) from 85.0% to 93.3%, and DT (Fig. 8) from 90% to 93%. This demonstrates that SHAP not only improves interpretability but also enhances predictive accuracy by prioritizing the most relevant features. Overall, the integration of SHAP reduces misclassification rates and strengthens the reliability of phishing detection models.

### Comparison with state-of-the-art methods

To critically assess the performance of the proposed method, we conducted a comparative evaluation against several state-of-the-art approaches, including classical ML models, deep learning architectures, hybrid frameworks, and large language model (LLM)-based detectors. Table 4 summarizes this comparison in terms of accuracy, precision, recall, and F1-score. Only studies reporting directly comparable metrics were included.

**Table 4:** Comparison of the Proposed RF+SHAP Model with State-of-the-Art Phishing Detection Approaches This table compares the proposed phishing detection model with existing state-of-the-art approaches. It includes models such as RF (baseline), SVM, CNN+LSTM, and DNN, showing the superiority of the proposed model across multiple performance metrics.

Model / Paper Name	Accuracy (%)	F1-Score (%)	Precision (%)	Recall (%)
Proposed RF + SHAP	97.0	97.3	97.0	97.6
RF (Baseline)^66^	96.25	96.2	97.6	98.3
SVM^67^	94.2	94.8	93.5	96.1
CNN+LSTM (IPDS)^68^	93.28	93.29	93.30	93.27
DNN^69^	92.89	92.21	92.75	93.07
LightGBM^70^	95.1	95.3	95.2	95.5
DeepSeek R1 Distill Qwen 14B Q8^71^	75	76	81	72

The proposed RF+SHAP model achieved 97.0% accuracy and an F1-score of 97.3%, surpassing traditional ML classifiers and deep learning-based methods. The baseline RF model performed competitively with 96.25% accuracy, but lacked the interpretability and feature transparency introduced by SHAP. Deep learning models, such as CNN+LSTM and DNN, achieved strong results in the range of 92–93%, but their higher computational costs and limited explainability make them less suited for lightweight, real-world deployments. More recent LLM-based detectors, such as DeepSeek R1 Distill, underperformed significantly with only 75% accuracy, indicating that large models trained for general purposes may not yet be optimized for phishing detection tasks. Overall, these results confirm that the proposed RF+SHAP framework not only achieves state-of-the-art performance but also addresses a critical research gap by providing explainability and resource efficiency. This balance of accuracy, interpretability, and practicality makes the approach particularly relevant for real-world cybersecurity infrastructures.

## Discussion

The experimental evaluation of multiple ML models for phishing detection provides clear evidence of the contribution of SHAP in enhancing both predictive performance and interpretability. Across all tested models, the integration of SHAP resulted in consistent improvements in accuracy, precision, recall, and F1-score. As shown in Table 4, the proposed RF+SHAP approach achieved 97.0% accuracy, outperforming the baseline RF at 96.25% and surpassing other state-of-the-art methods such as SVM and CNN+LSTM. These findings demonstrate that SHAP facilitates a more effective utilization of features and contributes to a tangible performance advantage. The most significant performance gain was observed in RF (Fig. 6), where accuracy increased from 90.0% without SHAP to 97.0% with SHAP. This improvement reflects SHAP’s capability to identify and prioritize the most discriminative features, which was also evident from the SHAP feature importance plots presented in the results. Similarly, the KNN model demonstrated improved balance between precision and recall when SHAP was applied, leading to fewer cases of legitimate websites being misclassified as phishing. This reduction in false positives, confirmed by the confusion matrices, is especially relevant for real-world deployment, where excessive false alarms can reduce user trust. The interpretability provided by SHAP further strengthens its role in phishing detection. Apart from predictive measures, the capacity to interpret model predictions is essential for cybersecurity professionals. SHAP attributes contribution scores to all features, thus demonstrating the features that influence predictions. The experiments indicated that URL-related features and domain indicators made the greatest contribution to classification results, consistent with prior research and validating model outputs for integrity. The findings allow security practitioners to better understand detection mechanisms and maximize defense mechanisms.

Algorithmic variability of performance was observed as well, with ensembling methods such as RF being the highest on average, but other classification methods, such as DT, SVM, and KNN, also showed concrete improvement when applying SHAP. This is proof of SHAP’s adaptability across model families, suggesting that ensembles would inherently be more precise, but SHAP’s interpretability and incremental gains in accuracy apply across a broad range of algorithms. The experimental outcome, thus, supports the extensive applicability of SHAP to phishing detection tasks. There are, however, certain boundaries to consider. The training and test data used is large and general, but phishing tactics are quickly developing, and new techniques emerging might not be included. This poses a question regarding generalizability to novel patterns, which might influence real-world performance. Moreover, the inclusion of SHAP into the workflow presented computational latency during experimentation. The calculation of SHAP values was observed to increase processing time, particularly for larger folds, which may restrict its applicability in scenarios requiring near real-time responses. Another source of variability lies in the sensitivity to hyperparameters and random initialization. Although five-fold cross-validation reduced this effect, some fluctuations were still observed, indicating that further optimization could stabilize outcomes. While SHAP improved both interpretability and performance, the experiments also revealed that interpretability alone does not guarantee overall model robustness. The models remain susceptible to adaptive phishing strategies and potential adversarial manipulation. Fairness and resilience against evolving threats are not addressed directly by SHAP explanations, meaning that interpretability must be considered alongside robustness and security-oriented evaluation metrics.

## Conclusion and future work

The integration of SHAP with supervised ML models improves both model interpretability and predictive performance in detecting phishing websites. In our experiments, the RF model combined with SHAP outperformed traditional models such as SVM, DT, and standard RF across all key performance metrics, including accuracy, precision, recall, and F1-score. SHAP enables the identification of the most influential features, allowing the model to focus on characteristics that strongly indicate phishing attempts and enhancing the transparency of decision-making processes. Compared to generic feature selection approaches, the proposed method more effectively highlights features with substantial impact on predictions, contributing to a reliable and robust phishing detection system. The five-fold cross-validation and evaluation across multiple supervised algorithms confirm the consistency of these results, showing that models incorporating SHAP not only achieve higher performance but also provide interpretable insights useful for cybersecurity practitioners.

Nevertheless, certain limitations remain. The dynamic and evolving nature of phishing attacks requires periodic retraining or adaptive mechanisms to maintain performance. The computational complexity of SHAP, particularly with very large datasets or deep architectures, can increase training and evaluation time. Furthermore, model generalizability is constrained by the dataset scope; testing primarily on structured URL-based features may not fully capture the diversity of phishing strategies, including those embedded in multimedia or multi-lingual platforms.

Building upon these findings, future research will explore several directions. First, expanding experimentation with larger, more diverse, and real-world datasets including multi-lingual and mobile phishing samples will help validate the scalability of the framework. Second, the development of lightweight SHAP-based variants or approximation techniques can improve computational efficiency, making the approach more suitable for low-resource environments such as IoT and edge devices. Third, integration with advanced deep learning architectures, such as transformer-based models, coupled with explainability tools, offers a promising avenue for combining high accuracy with interpretability. Fourth, adversarial robustness against phishing attempts that actively attempt to evade detection should be systematically studied. Finally, practical deployment and evaluation in real-world cybersecurity infrastructures, such as email gateways, browser plug-ins, or enterprise firewalls, will help assess usability and operational value.

Overall, the proposed approach demonstrates that combining SHAP with RF enhances phishing detection performance while ensuring transparency. By addressing the above limitations and extending research in the proposed directions, future studies can further advance the field toward highly accurate, interpretable, and resource-efficient phishing detection solutions suitable for dynamic and large-scale cybersecurity applications.

## Data Availability

The dataset used in this research is publicly accessible in the UCI Machine Learning Repository at https://archive.ics.uci.edu/dataset/327/phishing+websites. This benchmark dataset, provided by Mohammad et al., is commonly employed in phishing detection studies and supports reproducibility and comparability of results across studies.
